# The Effect of Big Data Analytics Capability on Competitive Performance: The Mediating Role of Resource Optimization and Resource Bricolage

**DOI:** 10.3389/fpsyg.2022.882810

**Published:** 2022-06-10

**Authors:** Bo Huang, Jianmin Song, Yi Xie, Yuyu Li, Feng He

**Affiliations:** ^1^School of Economics and Business Administration, Chongqing University, Chongqing, China; ^2^School of Economics and Management, Wuhan University, Wuhan, China; ^3^School of Economics and Management, Chongqing Normal University, Chongqing, China; ^4^Department of Tourism Management, Chongqing City Vocational College, Chongqing, China

**Keywords:** big data analytics capability, resource optimization, resource bricolage, competitive performance, PLS-SEM

## Abstract

Although big data analytics capability (BDAC) leads to competitive performance, the mechanism of the relationship is still unclear. To narrow the research gap, this paper investigates the mediating roles of two forms of resource integration (resource optimization and resource bricolage) in the relationship between two forms of BDAC [big data analytics (BDA) management capability and BDA technology capability] and competitive performance. Supported by Partial Least Squares-Structural Equation Modeling (PLS-SEM) and the cross-sectional survey data from 219 Chinese enterprises, the results show that the resource bricolage plays a significantly mediating role in the relationships between BDA management capability and competitive performance as well as in the relationship between BDA technology capability and competitive performance. Furthermore, the mediating effect in the former relationship is stronger than that in the latter relationship. Additionally, BDA technology capability only has a direct effect on resource bricolage, while BDA management capability has a stronger effect on resource optimization than that on resource bricolage. Finally, resource bricolage has a stronger impact on competitive performance than resource optimization. These findings contribute to understanding how enterprises could apply different forms of BDAC to other kinds of resource integration to achieve outstanding competitive performance.

## Introduction

Big data, defined as the vast amounts of data involving three characteristics of volume, velocity, and variety (Kauffman et al., 2012; Rana et al., 2021), is one of the most valuable resources for an enterprise to acquire competitive performance (Akter et al., 2016; Gupta and George, 2016). Therefore, big data analytics capability (hereinafter referred to as “BDAC”) is a vital capability to help enterprises reestablish new competitive advantages and achieve remarkable competitive performance (Wamba et al., 2017; Shahbaz et al., 2019; Mikalef et al., 2020; Awan et al., 2021a), due to that it can improve decision-making quality (Shamim et al., 2020; Awan et al., 2021b), redefine business logic (Ciampi et al., 2021; Zhu et al., 2022) and promote innovation (Mikalef et al., 2019). A business survey reports that several representative corporations, such as Airbnb, Amazon, and Netflix, have achieved unprecedented growth by BDAC (Shahbaz et al., 2020; Olabode et al., 2022).

Although there is no controversy over the direct effect of BDAC on competitive performance (Akter et al., 2016; Mikalef et al., 2020), its mechanism is still ambiguous, which prevents the positive effects from playing out to a greater extent (Günther et al., 2017; Wamba et al., 2017; Awan et al., 2021c). Recently, several mediators have been studied, such as dynamic capability (Mikalef et al., 2020), knowledge management (Ferraris et al., 2019), entrepreneurial orientation (Ciampi et al., 2021), and disruptive business model (Olabode et al., 2022). Nevertheless, researchers neglect the fact that a certain enterprise consists of a series of unique resources and their competitive performance largely depends on efficient integration and utilization of resources (Barney, 2001). Wielgos et al. (2021) also suggest that leveraging digital technologies to transform business is substantially an ongoing process which focuses on combining data, technology, and business to significantly change firm resources. A typical example is that Zhubajie, an internet platform company, used BDAC to dig out and integrate a large number of idle data resources to match more suppliers with more buyers, obtaining higher profits (Li et al., 2022). Unfortunately, current literature fails to advance knowledge on how BDAC create competitive performance. This study aims to address two research questions: *(1) Do two forms of resource integration (resource optimization and resource bricolage) play the mediating role between BDAC and competitive performance? (2) To what extent do the two forms of BDAC (BDA management capability and BDA technology capability) influence two forms of resource integration, resource optimization and resource bricolage, to achieve competitive performance?*

In order to address the question and respond to the call of filling the research gap (Günther et al., 2017; Wamba et al., 2017; Mikalef et al., 2019), the article establishes a theoretical framework from the perspective of resource-based theory. In the framework, BDAC as the ability to concentrate on extracting business opportunities from vast amounts of data is the antecedent variable and has two forms: BDA management capability and BDA technology capability. BDA management capability is an organizational capability to integrate core business operational functions, and BDA technology capability is a basic capability to guarantee data acquisition and development (Akter et al., 2016; Ferraris et al., 2019; Sun and Liu, 2020). Additionally, the theoretical framework includes resource integration as a mediator, which is a strategic behavior of various resource mobilization (Wiklund and Shepherd, 2009; Reymen et al., 2015). Generally, it also has two forms: resource optimization and resource bricolage (Desa and Basu, 2013). Resource optimization attaches importance to the efficient allocation of high-quality resources, which aims to complete the established opportunities or needs (Oliver, 1997; Desa and Basu, 2013; Lu and Guo, 2018). Resource bricolage refers to the process that enterprises take well advantage of existing undervalued, slack, or discarded resources at hand to save cost and develop new opportunities (Baker and Nelson, 2005; Duymedjian and Rüling, 2010; Desa and Basu, 2013). Furthermore, the dependent variable in the framework is competitive performance, which is commonly defined as the economic value of outperforming competitors (Barney and Clark, 2007; Awan, 2019). Competitive performance consists of financial performance and growth performance, both of which focus on sustainable development (Monferrer Tirado et al., 2019; Awan and Sroufe, 2022). Finally, PLS-SEM is employed to empirically test the hypotheses because its unique advantages in the estimation of models and its widespread use in the field of information system (Ali, 2021; Awan et al., 2021a).

This paper has two contributions. Primarily, this research finds out the vital mechanism of the resource bricolage rather than resource optimization in the relationship between BDAC and competitive performance. Specifically, resource bricolage plays a significant mediating role in the relationships of BDA management capability and BDA technology capability on competitive performance. Thus, this paper provides authentic evidence for answering the core question on “How does BDAC lead to competitive performance”. Although several studies have discovered some mediators, they overlook the fact that the competitive performance of a certain enterprise largely depends on effective resource integration and utilization (Wamba et al., 2017; Ciampi et al., 2021). This paper contributes to extending the exploration into the mechanism by demonstrating the effect of BDAC on competitive performance through resource bricolage.

Secondly, this study also narrows the gap in prior studies (Akter et al., 2016; Ferraris et al., 2019; Ciampi et al., 2021) which regard BDAC as a unidimensional construct in discussing the effect of BDAC on competitive performance. Comparing different mediating effects of resource bricolage in the pathway connecting BDA management capability with competitive performance as well as in the pathway connecting BDA technology capability with competitive performance, this study provides theoretically analysis for answering the question “To what extent do the two forms of BDAC influence which forms of resource integration in order to achieve competitive performance” (Mikalef et al., 2020). The results show that resource bricolage plays a stronger mediating role in the pathway connecting BDA management capability with competitive performance than that in the pathway connecting BDA technology capability with competitive performance. In other words, this paper contributes to understanding the special causal effects of different forms of BDAC on the relationships between different types of resource integration and competitive performance.

The rest of this article includes five parts: Section “Theoretical Background” presents the theoretical background. Section “Hypotheses Development” elaborates the research hypotheses. Section “Research Method” introduces the research method. Section “Results” accounts for the results. Section “Conclusion” discusses our findings, theoretical and managerial implications, limitations and future directions.

## Theoretical Background

### Resource-Based Theory

Resource-based theory provides an appropriate theoretical lens to investigate BDAC because it is born to be devoted to explaining the source of enterprises’ competitive advantages (Akter et al., 2016; Awan et al., 2021a). Specifically, the fundamental assumption of the theory is that an enterprise usually consists of a series of unique resources and capabilities due to resource immobility and its performance depends on what resources it possesses and how the resources are organized and utilized (Wernerfelt, 1984; Barney, 2001). Meanwhile, capabilities are intangible and the subsets of resources concentrate on the dynamics of resources and improving the productivity of resources (Barney and Clark, 2007; Akter et al., 2016). BDAC includes data resource identification, data storage, and data analytics, which represents the development of organizational intangible resources (Barney, 2001; Awan et al., 2021a). Whereas, resource optimization and resource bricolage focus on deploying other resources, which aims to increase the productivity of resources (Desa and Basu, 2013; Lu and Guo, 2018). They are the crucial sources of core competitiveness in a big data environment and can bring competitive performance.

### Big Data Analytics Capability

Previous studies have emphasized that realizing the business value of big data depends not only on the quality of data but also on the quality of data collection, data storage, and analysis process (Gupta and George, 2016; Ferraris et al., 2019). Mikalef et al. (2019) also argue that in the era of big data, data resources can be easily copied by other rivals, but the capability to configure and mine these data is difficult to be imitated and copied. Therefore, BDAC is “the next management innovation” (Akter et al., 2016; Gupta and George, 2016; Awan et al., 2021b; Ciampi et al., 2021). According to prior research, Ferraris et al. (2019) conceptualized the two forms of BDAC: BDA management capability and BDA technology capability. BDA management capability refers to the intangible ability to supports enterprises’ business decisions, consisting of BDA planning, investment, coordination, and control (Akter et al., 2016; Ferraris et al., 2019; Sun and Liu, 2020). Specifically, BDA management capability guarantees the interaction between data and the present model by optimizing decision models, cost-benefit analysis, input–output cost-effectiveness and finally improves the identification of potential market opportunities (Barton and Court, 2012; Woerner and Wixom, 2015; Sun and Liu, 2020).

The BDA technology capability is conceptually seen as the infrastructural module that cannot only support data acquisition and development but also upgrade the flexibility of the BDA platform, which involves three aspects: connectivity, compatibility, and modularity (Akter et al., 2016; Sun and Liu, 2020). Connectivity is reflected in the connection between different business units and different functions within the organization, such as the R&D department and customer management, supply chain management, and finance department (Ferraris et al., 2019; Sun and Liu, 2020). Compatibility reflects the information-sharing mechanism established to implement decisions, such as health codes during the pandemic. Modularity refers to allowing digital systems to add or optimize default models to ensure the flexibility of the BDA platform, such as periodic system updates (Akter et al., 2016).

### Two Types of Resource Integration: Optimization and Bricolage

Resource-based theory emphasizes that the allocation of various resources is the key to boosting the transformation of specific resources and capabilities into financial growth and competitive performance (Barney and Clark, 2007; Ciabuschi et al., 2012; Lu and Guo, 2018). Previous studies have put forward two typical forms of resource integration: resource optimization and resource bricolage (Desa and Basu, 2013). Resource optimization is “the process in which enterprises efficiently coordinate and allocates standardized high-quality resources to reach specific goals and demands” (Lu and Guo, 2018). Since enterprises realize the advantages and potential value in resources, they prefer to acquire these unique resources at high prices to exert the scale spillover effect of the high-quality resources by optimization (Barney, 2001). Therefore, resource optimization focuses on goal-oriented resource management (Desa and Basu, 2013).

Resource bricolage refers to “the process of making do by the combination of existing resources and saving costs,” concentrating on the effective utilization of undervalued, slack, and discarded resources (Desa and Basu, 2013; Senyard et al., 2014). On the one hand, bricolage aims to minimize resource costs and reduce the dependence on high-quality resources (Desa and Basu, 2013). On the other hand, bricolage is also an effective way to bring pioneering opportunities and capabilities because the bricolage can lead to the creative utilization of discarded resources (Duymedjian and Rüling, 2010). In other words, resource bricolage concentrates more on method-oriented resources management.

## Hypotheses Development

### Big Data Analytics Technology Capability, Resource Optimization and Bricolage

Prior studies have pointed out that BDA technology capability plays an important role in developing and deploying enterprises’ resources (Ferraris et al., 2019; Sun and Liu, 2020), which undoubtedly promotes resource integration. The distinctive feature of BDA technology capability is that it provides the enterprise with a flexible, dynamic, and integrated data support platform which not only enables a substantial number of unstructured data to be presented but also promotes a considerable amount of data from the database to the front end (Mikalef et al., 2020; Shamim et al., 2020; Sun and Liu, 2020). Since the data is readily accessible, enterprises can effectively monitor the whole production chain via digital technologies (Dubey et al., 2019), such as digital analysis of input and output, process supervision, and resource consumption. BDA technology capability can help enterprises to quantify when and to what extent both quality resources and undervalued resources generate business value and improve the utilization efficiency of resources (Sorescu, 2017; Lokshina et al., 2018).

Additionally, the connectivity of BDA technology capability could reduce the information asymmetry inside and outside the enterprise by connecting multiple participants (Wei et al., 2021), which not only broadens the scope to find superior resources but also reduces the searching cost of purchasing the resources (Ferraris et al., 2019). Once the cost of high-quality resources is significantly reduced, enterprises will pay more attention to using them to achieve scale effect, which will promote resource optimization. Furthermore, the connection between diverse stakeholders creates many potential opportunities and demands, making efficient use of those undervalued, slack, and discarded resources. Srinivasan and Arunasalam (2013) prove that BDA technology capability could elevate the performance of enterprises by reducing cost and improving the quality of patient care. More importantly, the compatibility and modularity of BDA technology capability can help enterprises to bridge the gap between resources and opportunities, and can inspire innovative approaches to matching high-quality resources with utilizing undervalued resources by modifying or reorganizing the existing business modules (Baert et al., 2016; Xie et al., 2020).

However, it is worth noting that the resource integration effect of BDA technology capability on resource bricolage is stronger e rather than that on resource optimization. In terms of reducing information asymmetry, BDA technology capability is significantly more effective in processing internal information than external information because interdepartmental data sharing and data authenticity are easier to achieve within a certain organization (Sun and Liu, 2020; Ranjan and Foropon, 2021). In other words, the management cost reduced by utilizing undervalued resources is more than the searching cost reduced by broadening superior resources. For instance, Zhu Bajie, a famous Internet company located in Chongqing, expanded its original business module of intellectual property by mining the data on its own platform, from the simple connection between supply and demand to the whole process of service. Meanwhile, from the perspective of resource possession, enterprises can rely on BDA technology capability to take full advantage of undervalued resources and get rid of the dependence on superior resources (Desa and Basu, 2013; Ciampi et al., 2021). Based on compatibility and modularity, enterprises are more likely to differently understand the potential value of undervalued and discarded resources as well as how to combine them in new ways to create value (Ferraris et al., 2019). Therefore, we propose that:

H1: BDA technology capability is positively associated with resource optimization.

H2: BDA technology capability is positively associated with resource bricolage.

H3: BDA technology capability has a stronger effect on resource bricolage than on resource optimization.

### Big Data Analytics Management Capability, Resource Optimization and Bricolage

Big data analytics management capability reflects the intangible ability of an organization to apply big data to implementing plan, investment, coordination and controlling activities (Akter et al., 2016; Ferraris et al., 2019; Sun and Liu, 2020). Differs from predominantly experience-oriented management capability, BDA management capability emphasizes efficient and quantitative management in organizational business based on data-driven perspective, which guarantees that enterprises have the ability to analyze the daily business and activities from a more comprehensive level, such as resource foundation, supply chain operation, consumer demand and the competitive environment (Akter et al., 2016; Anser et al., 2020; Shamim et al., 2020; Awan et al., 2021b). This management logic is of great significance in promoting resource acquisition and integration. More specifically, since BDA management capability pays more attention to data-driven quantitative decisions, it enhances some important soft power of enterprises. For example, data-driven culture greatly improves the rate and efficiency of resource transformation and integration (Chirico and Nordqvist, 2010; Shamim et al., 2020).

Secondly, BDA management capability expands the role of coordination attribute and control attribute in daily business, and facilitates the information sharing and knowledge spillover between different departments in enterprises (Akter et al., 2016; Ferraris et al., 2019; Awan et al., 2021a). This process not only helps enterprises to objectively understand the specific situation of resource consumption, but also may derive a new way of resource integration, which is of great of importance for enterprises to when and how adopt what type of resources. Thirdly, management capability driven by big data is also unique in identifying potential excellent partners, as the link between data of different sources makes some ambiguous relationships more explicit (Dubey et al., 2019), which ensures both stable access to quality resources and stable process of utilizing quality resources. As for those undervalued, slack and discarded resources, some new managerial innovations brought by BDA management capability provide a new path for them and revive their business value.

Since the positive effects of BDA management capability on resource optimization and resource bricolage are confirmed, this study also hypothesizes that the effect of BDA management capability on resource optimization is stronger than that on resource bricolage. BDA management capability is not as disruptive as BDA technology capability because it focuses more on taking advantage of data to reduce subjectivity and ambiguity in management (Gupta et al., 2019; Sun and Liu, 2020). When enterprises focus on improving the efficiency of resource utilization, they are more likely to concentrate on the efficiency of superior resources because superior resources are mature and competitive. On the contrary, enterprises will pay relatively little attention to the undervalued resources because the commercial value contained in them has not been proved before (Golroudbary et al., 2019). Additionally, from the perspective of resource attributes, the economies of scale generated by superior resources are generally higher than undervalued resources, which is also an important motivation for enterprises to employ BDA management capability to promote resource optimization. Thus, based on these arguments, we propose that:

H4: BDA management capability is positively associated with resource optimization.

H5: BDA management capability is positively associated with resource bricolage.

H6: BDA management capability has a stronger effect on resource optimization than on resource bricolage.

### Resource Optimization and Bricolage, and Competitive Performance

There is no doubt that the effective process of resource integration contributes to competitive performance improvement (Desa and Basu, 2013). As for resource optimization, as the scarcity of high-quality resources is not only an important basis for the unique competitiveness of enterprises but also important evidence to explain the source of Schumpeterian rents (Zahra et al., 2008; Ciabuschi et al., 2012), focusing on seeking and possessing of high-quality resources is conducive to deepening enterprises’ competition barriers and maintaining their existing competitive position. Some typical examples are high-tech companies like Apple, Huawei, and IBM, whose investment in innovation and R&D continues to increase, especially in terms of talent investment. In addition, the reorganization of superior resources tends to increase productivity and the economies of scale, which also leads to significant reduction in the unit cost (Desa and Basu, 2013; Lu and Guo, 2018). Furthermore, the optimization of quality resources can also stimulate innovation, especially disruptive innovation. In a nutshell, resource optimization breaks the traditional framework of resource utilization and develops a new solution in a novel way. ByteDance, for example, tried its best to facilitate the connection between the front end of product (consumer demand) and the back end of technology (product development), leading to the revolutionary product – TikTok.

On the contrary, resource bricolage is oriented to utilizing the undervalued, slack and discarded resources and achieving performance by reducing management costs (Baker and Nelson, 2005). More specifically, resources bricolage fully broadens the scope of the utilization of existing resources and makes these resources which originally do not generate value produce new value (Duymedjian and Rüling, 2010; Sonenshein, 2014). For example, Zhubajie mentioned above collected commissions by combining supply and demand information. More recently, however, it had broadened its business by mining and analyzing the vast amounts of data on the platform, making it profitable. When the undervalued resources without competitive advantages are fully utilized, the rapid flow of them can also reduce the related management costs, which is a competitive advantage compared with other competitors (Senyard et al., 2014). Similarly, resource bricolage can also enlighten innovation because of the creative use of undervalued, slack and discarded resources (Stenholm and Renko, 2016; Lu and Guo, 2018).

Although resource optimization and resource bricolage are all positively related to competitive performance, there are slight differences in the effects. Specifically, the competitive performance produced by resource bricolage is generally more distinguished than that produced by resource optimization. To begin with, the high-quality resources are often much scarcer than ordinary resources (Desa and Basu, 2013). Generally speaking, even though it can shape competitive performance by establishing competition barriers, enterprises have to invest huge amounts of money upfront and hence bear a heavy burden. Meanwhile, although quality resources can inspire disruptive innovation, the cycle is often very long and innovation is always full of many uncertainties, which is enough to explain why disruptive innovation is only reflected in a few products (Osiyevskyy and Dewald, 2015). What’s more, since digital technology has broken the boundary of resource acquisition, resources between enterprises are increasingly homogeneous. In other words, quality resources are no longer a kind of advantage (Lyytinen et al., 2016). Resource sharing has gradually become an important feature of modern business, which means that the role of matching superior resources to obtain competitive performance is gradually weakening. In more cases, enterprises are effective in utilizing homogeneous resources.

In short, leveraging the business value of undervalued, slack and discarded resources plays an increasing important role in resource mobilization. In most cases, the resources of enterprises will get depleted and what they can do is to do business based on necessity (Duymedjian and Rüling, 2010). Therefore, the barrier for them to adopt resource bricolage to improve performance is lower than resource optimization. For example, a great number of new ventures or SMEs rely on resource bricolage to achieve dramatic growth. Thus, we propose that

H7: Resource optimization is positively associated with competitive performance.

H8: Resource bricolage is positively associated with competitive performance.

H9: Resource bricolage has a stronger effect on competitive performance than does resource optimization.

**FIGURE 1:**
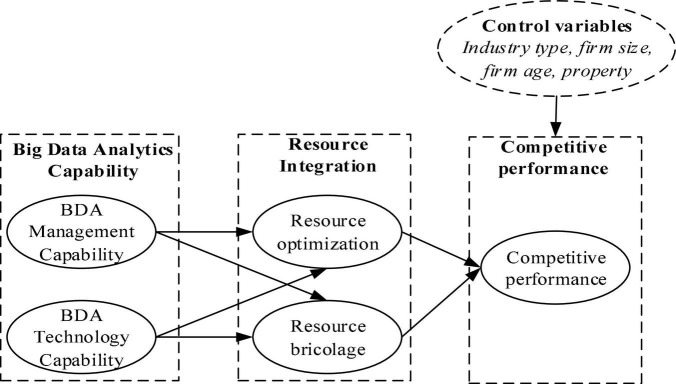
Research model.

Based on the above theoretical deduction, we put forward the hypothesized relationship in Figure 1.

## Research Method

### Data Collection

The data in this study was cross-sectional and collected by a method of self-administered questionnaire survey at multiple timepoints in China and the sample industries cover manufacturing, retailing, food service, IT, and so on. Why this method of survey is adopted is that it has several advantages in exploratory research and prediction theory testing (Straub et al., 2004), which greatly conforms to the research status of BDAC in the field of organization at present stage. Moreover, although all constructs involved in this paper are extracted from previous literature and there are still few studies to measure them by objective secondary indicators, the method used in this paper could make up for the defects of structural data.

The data was collected from Chinese enterprises because the innovation-driven development strategy of China motivates many companies to attach importance to BDAC investment. The respondents were randomly selected from the MBA and EMBA students of several universities located in Chongqing and Chengdu. The reason why we conduct such arrangements includes two points. First, Chengdu-Chongqing area has become one of the major regional centers under the support of national policies and strategic guidelines, which provides abundant enterprise samples for management research. Second, the majority of the MBA and EMBA students have a position as CEO, senior manager or department manager in different companies of different industries. Generally, they have a good understanding of the company’s strategy, business models and operating performance (Baskerville and Dulipovici, 2006; Yuan et al., 2021).

Before distributing questionnaires and collecting data, we drafted a full list of respondents with the help of the supervisors in MBA centers to ensure the implementation of survey. Moreover, we gathered and integrated the opinions of all participating enterprises before determining the final list of respondents and enterprises. In order to reduce the systematic errors caused by common method variance, the team members explained the original intention and purpose of scientific investigation to all respondents and promised to hide their personal information to guarantee anonymity before implementing the formal survey. We also took the errors into account in the design of measurement. The survey was implemented from March to May of 2021. At the first stage, we distributed paper questionnaires to students during class time with the teacher’s consent and collected the questionnaires after the class. By this way, we collected 242 questionnaires in which 135 questionnaires are valid with the completion rate of 55.78%. At the second stage, we distributed questionnaires to students through online tools such as email and WeChat. By this way, we collected 216 questionnaires in which 84 questionnaires are valid with the completion rate of 38.89%. Finally, we total distributed 458 questionnaires and collected 219 valid questionnaires with the completion rate of 47.82%. The sample details are shown in Table 1.

**TABLE 1:** Descriptive statistics of samples (*N* = 219).

Indexes	Category	Frequency	Per (%)	Indexes	Category	Frequency	Per (%)
Firm size (number of employees)	1–50	40	18.3%	Firm property	State-owned business	65	29.7%
	51–150	45	20.5%		Private enterprises	108	49.3%
	151–250	25	11.4%		Joint ventures	20	9.1%
	251–500	31	14.2%		WFOE	18	8.2%
	Above 500	78	35.6%		Others	8	3.7%
Industry type	Manufacturing	66	30.1%	Firm age	<1 year	7	3.2%
	Retailing	21	9.6%		1–4 years	39	17.8%
	Foodservice	20	9.1%		5–8 years	39	17.8%
	IT	31	14.2%		>8 years	134	61.2%
	Others	81	37.0%				

### Measurements

All scales adopted in the research are derived from validated measurements tools. Considering that the questionnaires are distributed in China, we choose Brislin’s (1980) “translation and back-translation” procedure to translate the English items into Chinese. All the items are rated on a 7-point Likert-type scale (*1 = strongly disagree, 7 = strongly agree*). All measurements can be found in Supplementary Material.

In order to guarantee the validity and suitability of the items for the Chinese context, we adopted a double-blind translation procedure to do a pretest by two professors and four doctoral candidates whose majors are business and management as well as two professionals. Considering the constructive feedback from the experts, we made minor alternations to enhance the overall readability of the items.

#### Independent Variable

Following prior studies on BDAC (Akter et al., 2016; Sun and Liu, 2020), BDA technology capability and BDA management capability were operationalized as two distinct forms of BDAC. BDA technology capability was measured with five items, while BDA management capability was conducted with six items (Sun and Liu, 2020). A sample item was “The rest of the offices are connected to the core central office for sharing analytics insights.”

#### Dependent Variable

Scholars have argued that the single financial index could not present the overall competitive advantages of firms. Following prior studies by Brinckmann et al. (2011) and Monferrer Tirado et al. (2019), we used six items to measure competitive performance, including growth performance and financial performance. A sample item was “The sales growth of our organization is relatively satisfactory.”

#### Mediator Variable

Following previous studies on resource integration (Senyard et al., 2009; Wiklund and Shepherd, 2009; Lu and Guo, 2018), we used both eight-item scales to measure resource optimization and resource bricolage. A sample item was “Our firm is constantly accumulating unique and high-quality resources.”

#### Control Variables

Following the literature (Lee and Tang, 2018; Smolka et al., 2018), this study selected industry, firm size, firm age, and firm property to ensure control variables.

### Statistical Techniques

We adopted Partial Least Squares Structural Equation Modeling (PLS-SEM) to test our hypotheses because of its remarkable advantages. Compared with Covariance-Based Structural Equation Modeling, PLS-SEM does not require large samples. On the contrary, PLS-SEM has outstanding advantages in estimating the models with small samples because its estimation is based on partial least squares rather than maximum likelihood estimation (Fornell and Bookstein, 1982; Marsh et al., 1998). Furthermore, similar to Covariance-Based Structural Equation Modeling, PLS-SEM can also synchronously take complex models and all path relationships between multiple constructs into account (Hair et al., 2011). Particularly, there are more indicators to evaluate PLS-SEM than to assess Covariance-Based Structural Equation Modeling. More importantly, PLS-SEM can effectively deal with both non-normal and normal sample data, which contributes to exploratory forecasting research (Fornell and Bookstein, 1982). In recent years, PLS-SEM has been widely used in organization behavior, strategy, marketing and entrepreneurship (Ali, 2021; Ciampi et al., 2021; Li et al., 2021). Therefore, it is suitable to use PLS-SEM to test the relationships between complicated constructs because the theoretical framework developed in this study includes multiple variables and paths.

### Common Method Variance

Some approaches were adopted to test the common method variance. First, we carried out Harman’s single factor test for all items (Podsakoff et al., 2012) and found that the contribution rate of cumulative variance of the first factor without rotation is 45.213% that revealed that no single factor accounts for more than 50% which is the standard value widely used in scientific research to suggest that there was no substantial common method variance. Second, we also integrated all items into a single factor to test the common variance according to Lisboa et al. (2011). The result showed that *χ^2^* = 1600.069, *df* = 405, *χ^2^/df* = 3.951, *CFI* = 0.717, *TLI* = 0.696, *RMSEA* = 0.116, *SRMR* = 0.081 and the other fitting indicators were higher than the threshold, which revealed that the fitness for single factor model was poor and then the common method variance was acceptable. Third, the variance inflation factor (VIF) was used to test the collinearity (Kock, 2015) and the result listed in Table 2 showed that all VIF values were less than 4, which suggested no evidence of common method variance in our data.

**TABLE 2:** The results of reliability of measurement model (*N* = 219).

Constructs	Items	SFL	*SE*	*t*-value*^a,b^*	SMC	VIF	α	C.R	ρ_A_*^c^*	AVE
BDA technology capability	BDAT01	0.812	0.029	28.144	0.659	2.205	0.888	0.918	0.889	0.691
	BDAT02	0.842	0.029	29.587	0.709	2.400				
	BDAT03	0.858	0.025	34.644	0.736	2.467				
	BDAT04	0.815	0.031	26.301	0.664	1.962				
	BDAT05	0.829	0.032	25.947	0.687	2.153				
BDA management capability	BDAM01	0.743	0.035	21.043	0.552	1.789	0.879	0.909	0.881	0.626
	BDAM02	0.800	0.030	26.366	0.640	2.011				
	BDAM03	0.813	0.028	29.516	0.661	2.189				
	BDAM04	0.850	0.021	41.588	0.723	2.508				
	BDAM05	0.708	0.047	14.822	0.501	1.629				
	BDAM06	0.823	0.025	33.016	0.677	2.242				
Resource optimization	REO01	0.779	0.028	27.423	0.607	1.981	0.895	0.917	0.896	0.614
	REO02	0.821	0.026	31.602	0.674	2.464				
	REO03	0.791	0.033	28.856	0.626	2.140				
	REO04	0.792	0.033	24.020	0.627	2.102				
	REO05	0.793	0.029	27.402	0.629	2.092				
	REO06	0.750	0.038	19.983	0.563	1.924				
	REO07	0.754	0.038	20.016	0.569	1.867				
Resource bricolage	REB01	0.777	0.036	21.517	0.604	1.801	0.865	0.899	0.866	0.597
	REB02	0.779	0.032	24.415	0.607	2.116				
	REB03	0.779	0.031	25.349	0.607	2.018				
	REB04	0.786	0.028	27.634	0.618	1.858				
	REB07	0.751	0.035	21.591	0.564	1.761				
	REB08	0.762	0.033	23.196	0.581	1.688				
Competitive performance (CP)	CP01	0.873	0.020	44.383	0.762	2.910	0.913	0.933	0.918	0.699
	CP02	0.873	0.018	47.369	0.762	3.419				
	CP03	0.859	0.020	42.333	0.738	3.501				
	CP04	0.785	0.041	19.092	0.616	2.458				
	CP05	0.856	0.021	41.003	0.733	3.135				
	CP06	0.761	0.044	17.245	0.579	1.780				

*SFL, standardized factor loading; SE, standard error; α, Cronbach’s alpha; C.R, composite reliability.*

*^a^Test-statistics are obtained by 5,000 Bootstrapping runs; SMC, square multiple correlations.*

*^b^Absolute t-values > 1.96 are two-tailed significant at 5%.*

*^c^Dijstra–Henseler’s rho_A; AVE, average variance extracted. As elaborated in Section “Statistical Techniques,” PLS-SEM can take complex models and all paths between multiple constructs into account simultaneously (Hair et al., 2011), so the CFA of each variable is not necessary.*

## Results

### Measurement Model Assessment

#### Validity and Reliability

To evaluate scale validity, we tested three indexes: standard factor loading (SFL), Cronbach’s alpha, and square multiple correlations (SMC). A rule of thumb for standard factor loading is recommended to be at least 0.70 (Fornell and Larcker, 1981; Hair et al., 2011). The results of SFL suggested that three items were below the standard of 0.70, including REO08, REB05, and REB06. After excluding these three items, all items were higher than the recommended threshold (see Table 2). The *t*-statistics of the standard errors were greater than 1.96, two-tailed *p* = 0.05, which confirmed that all the items-uni-dimensionality were statistically significant (Roldán and Sánchez-Franco, 2012). Moreover, all core constructs’ Cronbach’s alpha satisfied the recommended value of 0.70 (Bryman and Bell, 2011; Hair et al., 2011). Furthermore, SMC is also a common index to evaluate internal reliability and is recommended to be 0.36 or higher. As shown in Table 2, SMC values of all items were statistically greater than 0.36, which indicated that both the construct itself and each item had good reliability.

Also, this study employed component reliability (C.R), Dijkstra-Henseler’s rho (ρ_A_), and AVE to assess the reliability comprehensively (Hair et al., 2014), as shown in Table 2. All indices of C.R and ρ_A_ reached the standard of 0.70, supporting that all five constructs were acceptable. Finally, the AVE values of five variables all exceeded the standard value of 0.5 (see Table 2), which indicated that the convergence validity of the core dimensions selected in this study was relatively ideal.

#### Discriminant Validity

To evaluate discriminant validity, we undertook two common approaches (Hair et al., 2011). First, the square root of the AVE of each variable should be higher than the correlation coefficient of the row and column in which they were located (Fornell and Larcker, 1981). As shown in Table 3, all the AVE values reached this requirement, indicating that the constructs were adequately discriminated. Another criterion is heterotrait-monotrait (HIMT). The results confirmed that all HIMT values between constructs were below the threshold of 0.9 (Henseler et al., 2015; Ali, 2021). We, therefore, insisted that the discriminant validity of our model was acceptable.

**TABLE 3:** The results of convergence and discriminate validity (*N* = 219).

	1	2	3	4	5
(1) BDA management capability	**0.791**	0.855	0.649	0.799	0.809
(2) BDA technology capability	0.753**	**0.831**	0.558	0.712	0.659
(3) Competitive performance	0.585**	0.504**	**0.836**	0.648	0.578
(4) Resource bricolage	0.698**	0.627**	0.583**	**0.772**	0.823
(5) Resource optimization	0.720**	0.591**	0.530**	0.728**	**0.783**

*p < 0.05, **p < 0.01, ***p < 0.001. Diagonal elements are the square roots of the AVE. The elements that appeared in the lower left are the Pearson correlation coefficient between constructs. The elements that appeared in the upper-right are the HTMT values.

### Structural Model Assessment

To assess the structural model, the blindfolding procedure (omission distance = 7) was confirmed, which suggested the value of *Q*^2^ predictive relevance is greater than 0. The results showed that (see Table 4) values of *Q*^2^ were higher than 0, which indicated that the PLS-SEM path model received adequate in-sample power (Khan et al., 2018).

**TABLE 4:** Results of path analysis (*N* = 219).

Structural path	Path coefficients	Supported or not?	95% BCa confidence interval	Effects size (*f*^2^)
**Hypothesized links (direct effect)**				
BDA technology capability → Resource optimization	0.113	Not Supported	[−0.045, 0.265]	0.012
BDA technology capability → Resource bricolage	0.234**	Supported	[0.067, 0.383]	0.048
BDA management capability → Resource optimization	0.634***	Supported	[0.509, 0.770]	0.365
BDA management capability → Resource bricolage	0.521***	Supported	[0.380, 0.678]	0.240
Resource optimization → Competitive performance	0.225*	Supported	[0.046, 0.417]	0.037
Resource bricolage → Competitive performance	0.419***	Supported	[0.245, 0.588]	0.130
**Non-hypothesized links (control variables)**				
Firm size → Resource optimization	−0.065	Not supported	[−0.243, 0.123]	0.003
Firm size → Resource bricolage	−0.141	Not supported	[−0.306, 0.029]	0.016
Firm size → Competitive performance	−0.039	Not supported	[−0.235, 0.170]	0.001
Firm age → Resource optimization	−0.105	Not supported	[−0.266, 0.097]	0.008
Firm age → Resource bricolage	−0.034	Not supported	[−0.198, 0.143]	0.001
Firm age → Competitive performance	−0.096	Not supported	[−0.264, 0.158]	0.007
Firm property → Resource optimization	0.087	Not supported	[−0.050, 0.217]	0.007
Firm property → Resource bricolage	0.038	Not supported	[−0.100, 0.175]	0.001
Firm property → Competitive performance	0.002	Not supported	[−0.131, 0.126]	0.000
Industry → Resource optimization	−0.085	Not supported	[−0.231, 0.082]	0.007
Industry → Resource bricolage	−0.255***	Supported	[−0.376, −0.140]	0.066
Industry → Competitive performance	−0.094	Not supported	[−0.242, 0.067]	0.008
*R*^2^_(Resource optimization)_ = 0.523	*Q*^2^_(Resource optimization)_ = 0.296			
*R*^2^_(Resource bricolage)_ = 0.511	*Q*^2^_(Resource bricolage)_ = 0.281			
*R*^2^_(Competitive performance)_ = 0.364	*Q*^2^_(Competitive performance)_ = 0.235			

*p < 0.05, **p < 0.01, ***p < 0.001. BCa, bias-corrected and accelerated; R^2^, determination coefficients; Q^2^, predictive relevance of endogenous (omission distance = 7).

Furthermore, standardized root means square residual (SRMR) was considered to assess the overall model fit. The result demonstrated that SRMR was 0.058, below the threshold of 0.08 (Hair et al., 2014; Henseler et al., 2015), indicating that the overall model had a good degree of fit.

### Path Model Analysis

**FIGURE 2:**
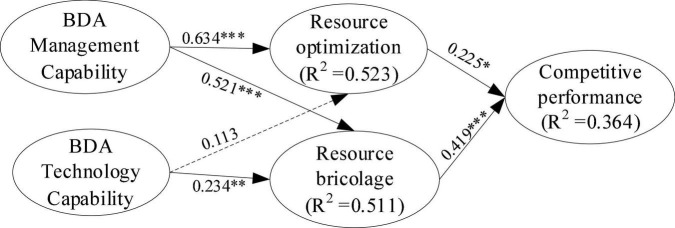
PLS path model. *p < 0.05, ^**^p < 0.01, ^***^p < 0.001.

We employed the PLS-SEM in SmartPLS 3.0 software to test our hypotheses. To test H1 and H2 which proposed that BDA technology capability was positively related to resource optimization and resource bricolage, the results (see Table 4 and Figure 2) suggested that BDA technology capability was only positively significantly related to resource bricolage (β = 0.234, *p* < 0.01), while the effect of BDA technological capability on resource optimization was not significant (β = 0.113, *p* > 0.05), demonstrating that H1 was not supported, but H2 was supported. To test H3, we compared the effect size (*f*^2^) and revealed that *f*^2^_(BDA technology capability → Resource bricolage)_ = 0.048 was greater than *f*^2^_(BDA technology capability → Resource optimization)_ = 0.012, that was, the effect of BDA technology capability on resource bricolage was stronger than it is on resource optimization, thus H3 was supported.

Also, the results (see Table 4 and Figure 2) demonstrated that BDA management capability was positively significant related to resource optimization (β = 0.634, *p* < 0.001) and resource bricolage (β = 0.521, *p* < 0.001), driving H4 and H5 were supported. To test H6, the results suggested that *f*^2^_(BDA management capability → Resource optimization)_ = 0.365 was greater than *f*^2^_(BDA management capability → Resource bricolage)_ = 0.240, supporting H6.

Lastly, we tested H7 and H8, which confirmed that resource optimization (β = 0.225, *p* < 0.05) and resource bricolage (β = 0.419, *p* < 0.001) were both positively associated with competitive performance, supporting H7 and H8. A comparative analysis was conducted to examine H9, and the results showed that *f*^2^_(Resource bricolage → Competitive performance)_ = 0.130 was greater than *f*^2^_(Resource optimization → Competitive performance)_ = 0.037, supporting H9.

The PLS path model could be considered a mediation model from Figure 2. To shed more light on this judgment, a non-parametric bootstrapping procedure was employed to explore possible indirect effects (Hayes and Preacher, 2013). As shown in Table 5, the results showed that under 95% confidence interval, only the indirect effect of resource bricolage was positively significant (β = 0.067, *p* < 0.05), and the confidence interval did not include 0 (LLCI = 0.013, ULCI = 0.145). However, the confidence interval for the impact of resource optimization included 0 (β = 0.009, *p* > 0.05; LLCI = −0.011, ULCI = 0.051). Thereby, resource bricolage played a mediating role in the relationship of BDA technology capability to competitive performance, while the mediating role of resource optimization was not supported.

**TABLE 5:** Mediation analysis results (*N* = 219).

	Total effect on competitive performance	Direct effect on competitive performance	Indirect effects on	

			Through resource optimization	Through resource bricolage
BDA technology capability	0.145	0.069	0.009 [−0.011, 0.051]	0.067* [0.013, 0.145]
BDA management capability	0.476***	0.273*	0.053 [−0.065, 0.181]	0.151* [0.046, 0.282]

*p < 0.05, **p < 0.01, ***p < 0.001. [] is 95% BCa confidence interval; bootstrapping set is 5,000.

Similarly, the mediating effect of resource bricolage in the relationship of BDA management capability on competitive performance was supported, since the indirect effect was positively significant (β = 0.151, *p* < 0.05; LLCI = 0.046, ULCI = 0.282), while resource optimization did not mediate this causal relationship (β = 0.053, *p* > 0.05; LLCI = −0.065, ULCI = 0.181). All the empirical results were present in Table 5.

Finally, the mediating effect of resource bricolage is stronger in the relationship between BDA management capability and competitive performance (β = 0.151, *p* < 0.05) than it between BDA technological capability and competitive performance (β = 0.067, *p* < 0.05).

### Endogeneity Testing

Endogeneity reflects the possible bias in causal studies, which is generally manifested in omitted variables, measurement errors, sample bias, and reverse causality (Ullah et al., 2018; Ali, 2021; Liu et al., 2021; Soluk et al., 2021). Although endogeneity has been widely emphasized in those studies based on secondary data, such as time-series data or panel data, it has not been fully tested in studies based on survey data. Recently, some pioneer scholars have demonstrated that endogeneity should be reported when using survey data (Ali, 2021; Liu et al., 2021; Soluk et al., 2021; Wielgos et al., 2021). To address the potential endogeneity in this study, we followed the research step of Ali, 2021 and Ullah et al. (2021), which adopted a two-step Heckman procedure to test possible endogenous bias. Table 6 showed that all regressions remained with the same statistical significance as PLS path model results, which indicated that our main regression models had not been affected by endogeneity.

**TABLE 6:** Endogeneity test (*N* = 219).

	Path coefficients	*SD*	*P*-value	*Z*	Conclusion
BDA management capability → Resource optimization (selection DV = Resource bricolage; IV = BDA technology capability)	0.597***	0.039	0.000	15.28	Not different
BDA management capability → Resource bricolage (selection DV = Resource optimization; IV = BDA technology capability)	0.567***	0.040	0.000	14.33	Not different
BDA technology capability → Resource optimization (selection DV = Resource bricolage; IV = BDA management capability)	0.419***	0.039	0.000	10.80	Not different
BDA technology capability → Resource bricolage (selection DV = Resource optimization; IV = BDA management capability)	0.437***	0.037	0.000	11.83	Not different
Resource optimization → Competitive performance (selection DV = Resource bricolage; IV = BDA technology capability)	0.695***	0.076	0.000	9.15	Not different
Resource bricolage → Competitive performance (selection DV = Resource optimization; IV = BDA management capability)	0.769***	0.074	0.000	10.43	Not different

*p < 0.05, **p < 0.01, ***p < 0.001. DV, dependent variable; IV, independent variable; SD, standard deviation.

## Conclusion

### Discussion of Results

First, this study proves the significant mediating effect of resource bricolage on the relationship between BDA management capability and competitive performance as well as the relationship between BDA technology capability and competitive performance. The empirical results demonstrate that enterprises could improve competitive performance by utilizing undervalued resources through resource bricolage (Ge et al., 2016). This finding is supported by Wielgos et al. (2021) who believe that BDAC can change the resource position of the enterprise, which will result in outstanding competitive performance. Therefore, this article contributes to filling the gap because previous studies neglected to investigate the mechanism of resource bricolage from a more basic perspective (Wamba et al., 2017; Zhu et al., 2022).

Second, this paper confirms that resource bricolage has a stronger mediating effect on the relationship between BDA management capability and competitive performance than that on the relationship between BDA technology capability and competitive performance. This finding reveals the different indirect effects of resource bricolage which are ignored by previous studies when exploring the causal link between BDAC and competitive performance (Wamba et al., 2017; Awan et al., 2021a). This paper also contributes to narrowing the gap because previous studies did not discuss the mechanism sufficiently from the two forms of BDAC (Ferraris et al., 2019; Ciampi et al., 2021).

Third, this research discovers that BDA technology capability only has a positive effect on resource bricolage, while the effect of BDA technology capability on resource optimization is not supported. This finding demonstrates that BDA technology capability is more conducive to improving the utilization of undervalued resources, which responds the proposal of Sun and Liu (2020) and Wielgos et al. (2021). This paper provides a theoretical guidance to improve the efficiency of utilizing undervalued resources via BDA technology capability.

Fourth, this paper finds that BDA management capability is positively related to both resource optimization and resource bricolage and the effect of BDA management capability on resource optimization is stronger than that on resource bricolage. This finding is supported by Mikalef et al. (2020) which contributes to overall understanding the special direct effects of BDA management capability on optimization and resource bricolage (Shamim et al., 2020; Zhu et al., 2022).

Fifth, this study demonstrates that the positive effects of resource optimization and resource bricolage on competitive performance and the effect of resource bricolage on competitive performance is stronger than the effect of resource optimization on competitive performance. This finding is similar to the result discovered by Desa and Basu (2013) who underlined the unique role of resource bricolage. This conclusion helps future study to understand the elevating effects of resource optimization and resource bricolage on performance. which is overlooked by previous studies in the era of big data (Desa and Basu, 2013; Lu and Guo, 2018).

### Managerial Implications

First, enterprises should pay more attention to utilizing undervalued resources by resource bricolage especially when they want to use BDAC to achieve remarkable performance in intense local and global competition. Specifically, managers and technicians are supposed to use digital technologies which include popular big data tools and cloud computing to dig out the potential value and business opportunities contained in undervalued resources. Furthermore, BDAC can drive enterprises to conduct digital supervision of resources to detect potential needs as quickly as possible.

Second, compared with BDA technology capability, enterprises should pay more attention to the construction and cultivation of BDA management capability in order to better expand the potential positive effect of undervalued resources on performance (Senyard et al., 2014). Therefore, enterprises should build data-driven culture (Li et al., 2021), promote digital communications between departments (Xie et al., 2020) and strengthen data sharing in interdepartmental cooperation (Sun and Liu, 2020). These measurements will be highly beneficial for achieving digital management of high-quality resources.

As a typical example, Zhubajie can be used to understand how an enterprise take advantage of BDAC to improve performance through the mediating path of resource bricolage. The company accumulated a large amount of data on buyers and suppliers in early commercial activities, but the lack of capability to analyze the data left the data in cold databases (Li et al., 2022). With the popularization and application of data technologies, Zhubajie began to pay more and more attention to mining and analyzing the platform background data which was considered as idle resource before to identify potential business opportunities and demands. Today, the company has developed to be a company who concentrates on data analytics and makes billions of dollars a year by analyzing backend data (Li et al., 2022).

### Limitations and Future Research

Like other empirical papers, there are also several limitations in the present research. First of all, there are still some errors in survey data because of the subjectivity in self-reported scales and questionnaires, such as homologous variance, which is generally remained in related research (Shamim et al., 2020). Even though considerable efforts are made to ensure the quality of survey data, such as hiding the personal information of the respondents and assessing the common method variance, subjective and objective errors are still inevitable. Multi-method measurement is of great advantage to solve the problem. Therefore, future research can introduce some new methods to reduce subjective errors of data, such as situational experiments and quantum computing (Soluk et al., 2021; Awan et al., 2022). Secondly, compared with longitudinal data, cross-sectional data still has some limitations on testing the hypotheses. Although several works that tried to test the robustness of the data collected by questionnaire have been done to rule out potential alternative explanations, future researches still should try their best to adopt case study or longitudinal tracking data to enhance the robustness of the findings (Ciampi et al., 2021; Li et al., 2021). Thirdly, this study only explained the special mechanism of the relationship between BDAC and competitive performance by considering resource integration. However, there may be other mechanisms worth of investigation, such as strategic orientation and alliance modes (He et al., 2020; Ciampi et al., 2021). Thus, future research could pay more attention to that. Fourth, this study did not spare no effort to take some important moderating variables into account, such as TMT supporting and environmental dynamic (Wamba et al., 2017). So future research can deeply explore the boundary conditions. Finally, compared with panel data, the sample size of this study is still relatively small, which provides a direction for researchers to expand the data range of samples to enhance the robustness of conclusions.

## Data Availability Statement

The raw data supporting the conclusions of this article will be made available by the authors, without undue reservation.

## Author Contributions

BH wrote the manuscript and mentoring. JS planned the study and wrote the manuscript. YX analyzed the data and reviewed the manuscript. YL provided the methodology. FH collected the data. All authors contributed to the article and approved the submitted version.

## Conflict of Interest

The authors declare that the research was conducted in the absence of any commercial or financial relationships that could be construed as a potential conflict of interest.

## Publisher’s Note

All claims expressed in this article are solely those of the authors and do not necessarily represent those of their affiliated organizations, or those of the publisher, the editors and the reviewers. Any product that may be evaluated in this article, or claim that may be made by its manufacturer, is not guaranteed or endorsed by the publisher.
