# Supplementary material for: The Effect of Big Data Analytics Capability on Competitive Performance: The Mediating Role of Resource Optimization and Resource Bricolage
Source: Front Psychol. 2022 Jun 10;13:882810. doi: 10.3389/fpsyg.2022.882810 (PMC9226484; doi:10.3389/fpsyg.2022.882810)
Supplement: Supplementary file 1 [file Table_1.docx]

# APPENDIX

**Scale Items for Key Constructs**

To what extent do you agree with the following statements?

(*1=strongly disagree; 7=strongly agree*)

| **Big data analytics capability** (Akter et al., 2016; Sun and Liu, 2020) | | | | | | | | |
| --- | --- | --- | --- | --- | --- | --- | --- | --- |
| BDA technology capability | —The rest of offices are connected to the core central office for sharing analytics insights. | 1 | 2 | 3 | 4 | 5 | 6 | 7 |
|  | —Our organization utilizes open systems network mechanisms to boost analytics connectivity. | 1 | 2 | 3 | 4 | 5 | 6 | 7 |
|  | —Software applications of our organization can be easily used across multiple analytics platforms. | 1 | 2 | 3 | 4 | 5 | 6 | 7 |
|  | —Employees can access all platforms through the company's user interface. | 1 | 2 | 3 | 4 | 5 | 6 | 7 |
|  | —To meet the various needs of data analysis, our organization will adjust its internal process system. | 1 | 2 | 3 | 4 | 5 | 6 | 7 |
| BDA management capability | —We continuously examine innovative opportunities for the strategic use of business analytics | 1 | 2 | 3 | 4 | 5 | 6 | 7 |
|  | —We perform business analytics planning processes in systematic ways. | 1 | 2 | 3 | 4 | 5 | 6 | 7 |
|  | —When we make business analytics investment decisions, we estimate the effect they will have on the productivity of the employees’ work. | 1 | 2 | 3 | 4 | 5 | 6 | 7 |
|  | —When we make business analytics investment decisions, we project how much these options will help end users make quicker decisions. | 1 | 2 | 3 | 4 | 5 | 6 | 7 |
|  | —In our organization, information is widely shared between business analysts and line people so that those who make decisions or perform jobs have access to all available know-how. | 1 | 2 | 3 | 4 | 5 | 6 | 7 |
|  | —In our organization, the responsibility for analytics development is clear. | 1 | 2 | 3 | 4 | 5 | 6 | 7 |
| **Resource integration** (Senyard et al., 2009; Wiklund and Sheperd, 2009; Lu and Guo, 2018) | | | | | | | | |
| Resource optimization | —In order to better accomplish the project, our firm can develop a planning about resource mobilization. | 1 | 2 | 3 | 4 | 5 | 6 | 7 |
|  | —Our firm is constantly accumulating unique and high-quality resources. | 1 | 2 | 3 | 4 | 5 | 6 | 7 |
|  | —In order to better accomplish the project, our firm often replenish the necessary new resources. | 1 | 2 | 3 | 4 | 5 | 6 | 7 |
|  | —Our firm enables to make full use of high-quality resources to innovate new productions or services. | 1 | 2 | 3 | 4 | 5 | 6 | 7 |
|  | —Our firm generally concentrates on improving resources flow through promoting internal exchanges and cooperation. | 1 | 2 | 3 | 4 | 5 | 6 | 7 |
|  | —Our firm absorbs necessary external resources in the process of external communication and cooperation. | 1 | 2 | 3 | 4 | 5 | 6 | 7 |
|  | —Our firm often summarizes our resource mobilization to improve its utilization in the future. | 1 | 2 | 3 | 4 | 5 | 6 | 7 |
|  | —When faced with resources constraint, our firm would consider outsourcing part of the project. | 1 | 2 | 3 | 4 | 5 | 6 | 7 |
| Resource bricolage | —Faced with environmental impact, our firm has found a feasible solution using existing resources. | 1 | 2 | 3 | 4 | 5 | 6 | 7 |
|  | —Compared with competitors, our firm is more willing to utilize existing resources to meet challenges. | 1 | 2 | 3 | 4 | 5 | 6 | 7 |
|  | —Our firm respond to new problems with whatever resources they have available. | 1 | 2 | 3 | 4 | 5 | 6 | 7 |
|  | —Our firm focuses on the utilization of undervalued, slack or discard resources to meet new challenges. | 1 | 2 | 3 | 4 | 5 | 6 | 7 |
|  | —When faced with a new problem, our firm always believes that it can find a solution and take action. | 1 | 2 | 3 | 4 | 5 | 6 | 7 |
|  | —Our firm often faces unexpected new challenges when combining existing undervalued resources. | 1 | 2 | 3 | 4 | 5 | 6 | 7 |
|  | —Our firm has achieved a viable solution through the existing resources integration. | 1 | 2 | 3 | 4 | 5 | 6 | 7 |
|  | —Our firm has integrated resources not originally devoted to the task to accomplish new challenges. | 1 | 2 | 3 | 4 | 5 | 6 | 7 |
| **Competitive performance** (Monferrer Tirado et al., 2019; Brinckmann, 2011) | | | | | | | | |
|  | －The sales growth of our organization is relatively satisfactory. | 1 | 2 | 3 | 4 | 5 | 6 | 7 |
|  | －The market share growth rate of our organization is relatively satisfactory. | 1 | 2 | 3 | 4 | 5 | 6 | 7 |
|  | －The growth rate of new employees is still satisfactory. | 1 | 2 | 3 | 4 | 5 | 6 | 7 |
|  | －The market share of our organization is still relatively satisfactory. | 1 | 2 | 3 | 4 | 5 | 6 | 7 |
|  | －The rate of return on investment of our organization is still satisfactory. | 1 | 2 | 3 | 4 | 5 | 6 | 7 |
|  | －The profit level of our organization is still satisfactory. | 1 | 2 | 3 | 4 | 5 | 6 | 7 |
